# Lignin for Bioeconomy: The Present and Future Role of Technical Lignin

**DOI:** 10.3390/ijms22010063

**Published:** 2020-12-23

**Authors:** Adam Ekielski, Pawan Kumar Mishra

**Affiliations:** 1 Department of Production Engineering, Warsaw University of Life Sciences, 02-776 Warsaw, Poland; adam_ekielski@sggw.pl; 2 Faculty of Business and Economics, Mendel University in Brno, 61300 Brno, Czech Republic

**Keywords:** lignin, technical lignin, self-assembly, lignin nanoparticles, lignin, bioeconomy

## Abstract

Lignin, the term commonly used in literature, represents a group of heterogeneous aromatic compounds of plant origin. Protolignin or lignin in the cell wall is entirely different from the commercially available technical lignin due to changes during the delignification process. In this paper, we assess the status of lignin valorization in terms of commercial products. We start with existing knowledge of the lignin/protolignin structure in its native form and move to the technical lignin from various sources. Special attention is given to the patents and lignin-based commercial products. We observed that the technical lignin-based commercial products utilize coarse properties of the technical lignin in marketed formulations. Additionally, the general principles of polymers chemistry and self-assembly are difficult to apply in lignin-based nanotechnology, and lignin-centric investigations must be carried out. The alternate upcoming approach is to develop lignin-centric or lignin first bio-refineries for high-value applications; however, that brings its own technological challenges. The assessment of the gap between lab-scale applications and lignin-based commercial products delineates the challenges lignin nanoparticles-based technologies must meet to be a commercially viable alternative.

## 1. Introduction

**Figure 1:**
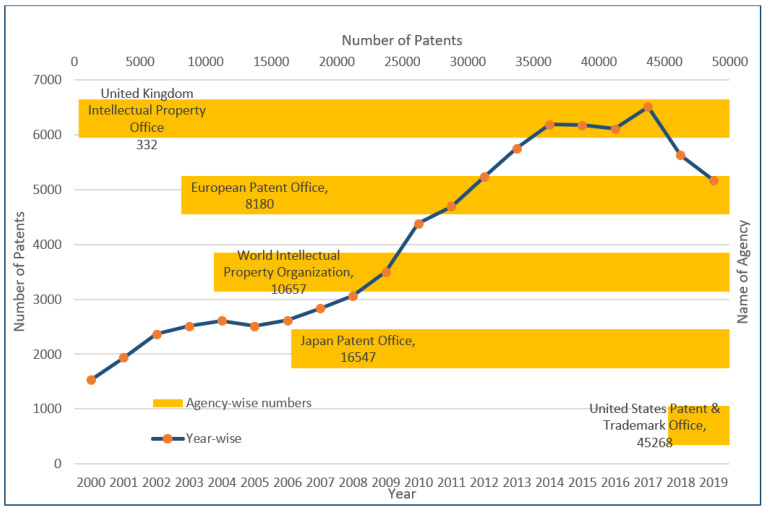
Number of patents registered in different agencies and the number of total patents in the duration of 2000–2019 (Results were obtained from the Scopus database using the keyword “lignin”).

Technical lignin refers to the “native-lignin” or “proto-lignin” derivative obtained as the result of the delignification process of the lignocellulosic-biomass [1]. The technical lignin structure can vary with the process and chemical reactions utilized in the biomass/starting material treatment [2]. Traditionally, technical lignin has been obtained as a by-product of paper mills wherein the revenue is primarily generated by the cellulose-based products, and therefore every process has been optimized to maximize the qualitative and quantitative yield of the cellulose [3]. The recent progress in technology and the arrival of “lignin-centric biorefinery” or “lignin-first biorefinery” is a welcomed addition to the biomass treatment strategies. It has renewed the interest in lignin from “valorization” to “application” approaches [4,5,6,7]. It has also provided an impetus to the search for structurally homogeneous lignin that can be easily applied in efficient and scalable technology for lignin-based reagents production [8]. The relatively new addition in lignin-based clean ( biocompatible and non-toxic) products include nanomaterials and those used in biomedical applications [9,10,11]. As presented in Figure 1, a multi-fold rise in the number of patents registered in the Scopus database in the period of the last 20 years (2000–2019) can be seen. The maximum number of patents were registered in the United States Patent and Trademark Office (45,268), followed by the Japan Patent Office (16,547), the World Intellectual property organization (10,657), the European Patent office (8180) and the United Kingdom Intellectual Property office (332). These results were obtained from the Scopus database with the keyword “lignin”. It reflects the commercial relevance of lignin-based research and development of technology for its high–value utilization.

**Figure 2:**
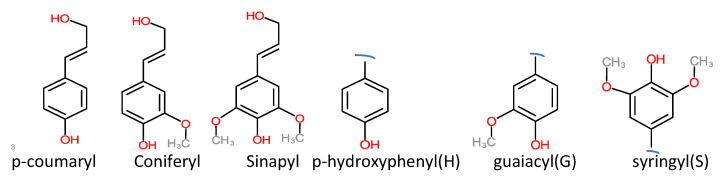
Common monomers of lignin.

**Figure 3:**
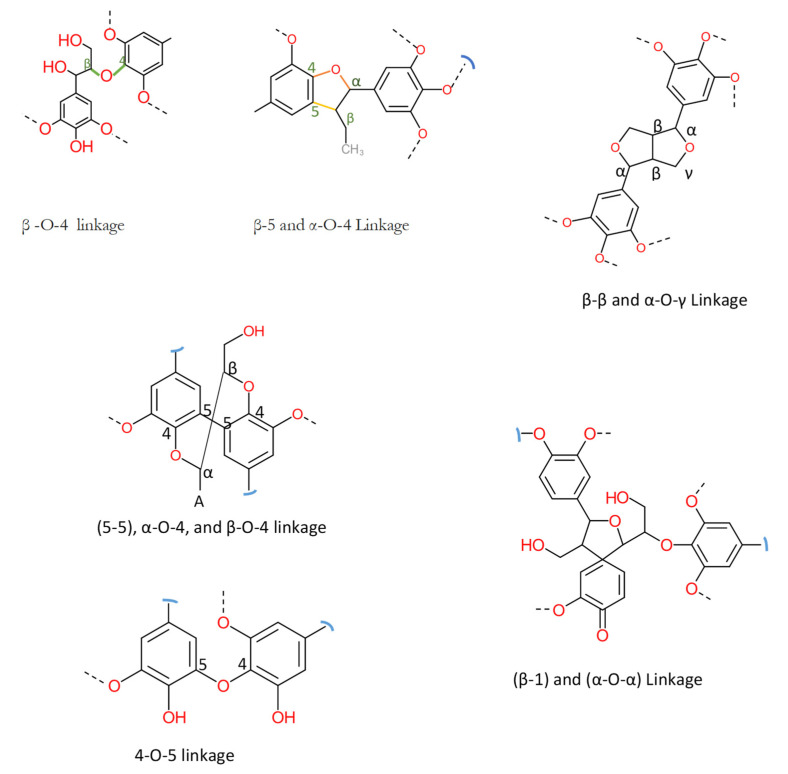
Common linkages found in lignin [18].

Technical lignin is derived mainly from lignocellulosic biomass, and therefore its structure directly depends on the source and method of extraction. Once the lignin (protolignin/native lignin) leaves the cell wall (extracted), its structure completely changed; however, monomers can still be identified. The lignin monomers include *p*-coumaryl alcohol, coniferyl alcohol, and sinapyl alcohol, bearing *p*-hydroxyphenyl (H), guaiacyl (G), and syringyl (S) units, as represented in Figure 2. The source-dependent variation in lignin can be observed in herbaceous crop-based (rich in H units), gymnosperm/softwood (lacking S units), and angiosperm/hardwood (rich in G and S) lignin [1,12]. The variation in monomers composition is supported by variation in nature and the extent of chemical bonding. Different bonds observed in lignin, along with their numbering system, are presented in Figure 3. The β-O-4 linkage represents the most dominant linkage of all three (softwood, hardwood, and grasses) types of sources; however, their comparative content follows the order grasses > hardwood > softwood. The two diastereomers, the erythro and threo forms of β-O-4 linkage, can be found in nature. The softwood lignin has approximately the same amount of both forms; the erythro form is prevalent in hardwood lignin. The quantification of the two forms (ratio and total amount) of these two forms can be done by ozonation that leads to the formation erythronic (from erythro form) and threonic (from threo form) acid, respectively [13]. The β-O-4 linkage is also the most easily affected bonding in the lignin by different treatment methods. Despite the relatively lesser number of β-O-4 linkage, the softwood lignin is primarily composed of coniferyl alcohol, highly condensed and a higher number of C-C bonds, 5′ linkages, β-β, and β-5 bonds, cross-linking, and branching. These properties make softwood challenging to degrade and relatively more resistant to chemical treatments (Table 1). Another aromatic material (not a lignin derivative) found in pulp analysis is termed pseudolignin. It consists of aliphatic and aromatic groups (methoxyl, carboxyl, and carbonyl groups) of non-lignin origin. Pseudolignin has also been observed in pulp obtained by steam explosion and dilute acid hydrolysis. [14,15]. It has been suggested that its formation is favored by low pH, high temperature, longer treatment time, and acid strength. The mechanism of pesudolignin formation has been suggested as repolymerisation of decomposition products of the sugars [16,17]. However, the pseudolignin formation is a different aspect of biomass delignification, and a detailed discussion on it is out of the scope of this manuscript.

In the case of technical lignin, the generalized information about lignin is not sufficient. The “technical lignin” specific data is needed to manipulate the material at molecular/nanoscale material for its utilization in high-value applications. Unfortunately, unanimously accepted data about technical lignin molecular characteristics is still missing [13,19]. It is due to the fact that even for the same method such as nuclear magnetic resonance (NMR) and size exclusion chromatography (SEC), the data varies depending on the chosen parameters. Several excellent reviews on lignin properties and applications have been published recently [20,21,22,23,24,25]. However, this review builds upon the fact that papermill-based technical lignin is still a standard and abundantly available raw material; therefore, successful technology must be built on its coarse but scalable properties. Different technical lignin (commercially available), their characteristics, and reported studies have been critically reviewed to develop a coarse level of generalized understanding. The recently reported (last 3 years) technical lignin nanoparticle and industry-specific applications have been studied along with marketed products. The gap between the lab-scale suggested application and marketed formulations has been used to underline lignin utilization challenges. Additionally, the future potential and challenges for technical lignin in the high-value applications have been covered for future applications.

### Classification of Technical Lignin

**Figure 4:**
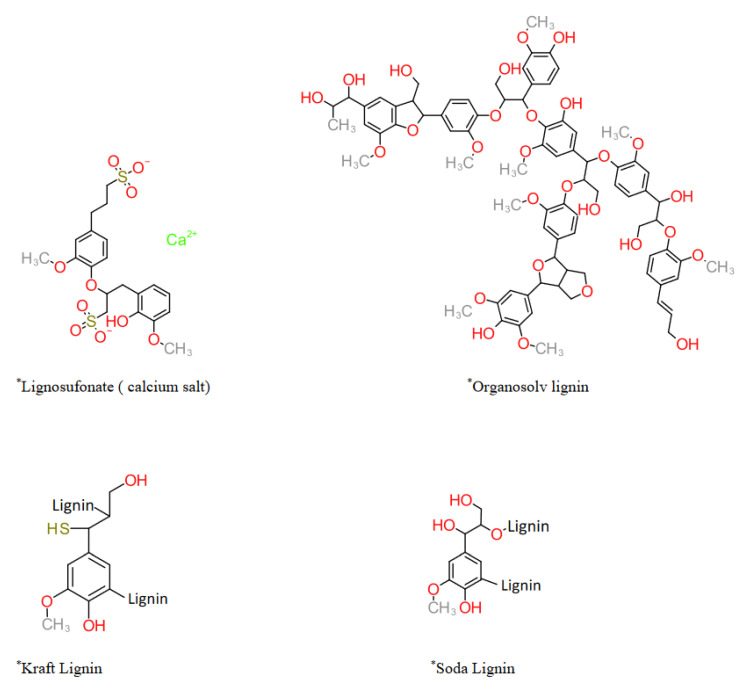
Common technical lignins (* simplified and representative structures).

The overall classification of lignin generally follows the criteria of the delignification/biomass treatment approach. Especially for technical lignin, a number of variations in pretreatment and/or lignin precipitation methodologies can be classed in the same categories. These classes also represent the changes in delignification and precipitation methods with time, leading to improved technology and, therefore, the final product. As shown in the following Table 2, several types of lignin can be obtained by the same broader class of treatment, such as Kraft lignin. Every delignification approach modifies the structure of lignin in its specific way, and it can be observed in the generalized representative structure of lignin in Figure 4. In addition to the generalized structure, treatment-based variation in nature and extent of C-C bond cleavage, degree of condensation, molecular weight, and quantitative and qualitative variations in functional groups of the obtained technical lignin are also important. These also directly affect the potential applications of technical lignin. For example, Kraft lignin contains covalently bonded sulfur species that constitute major impurities that limit and direct the further applications and valorization approaches (sulfur is known as poison for many metal-containing catalysts used for depolymerization). On the other hand, lignosulfonate has the sulfonate group on the aromatic ring that endows water-solubility. Additionally, their colloidal, surfactant and adhesive properties are due to the high density of functional groups. It is due to specific bond breakage caused by particular solvent-biomass interaction and the underlying mechanism. These aspects are discussed in detail in the following section.

## 2. Kraft Lignin

**Figure 5:**
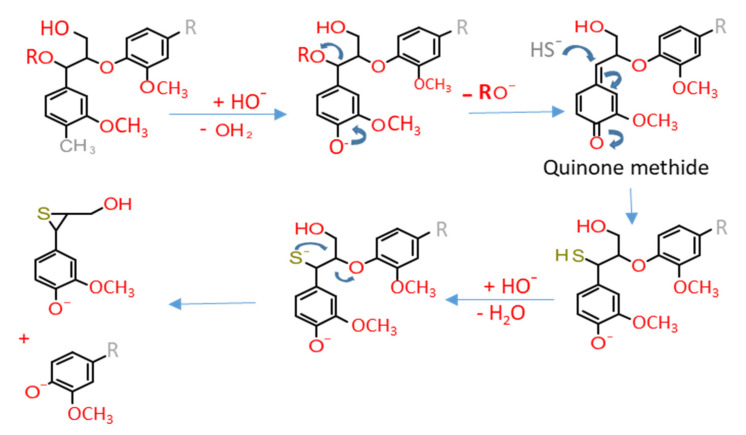
Cleavage of phenolic β-O-4 linkage during the kraft process [84]. Arrows represent reactions.

The “kraft”; a German term for “strength”, is used because of the superior strength of the resulting paper. It was discovered in 1879 by Carl F. Dahl (U.S. patent issued in 1884) [26]. The Kraft lignin is a major by-product of the kraft pulping process that utilizes a mixture of hot water (155–175 °C), sodium hydroxide (NaOH), and sodium sulfide (Na_2_S, also called as white liquor). During the pulping process, the dissolution of phenolic lignin occurs via ionization of phenolic groups (due to alkaline pH) and an unstable intermediate quinone methide (Figure 5). It is followed by the reaction of bisulfide ions (strong nucleophiles) with quinone methide and finally cleaving the β–aryl ether linkage [83,84]. Common nucleophiles participating in the kraft process are HO^−^ < HS^−^<S^2−^ in increasing order of nucleophilicity that attack the electron-deficient centers of lignin molecules and lead to the dissolution of lignin in processing liquor [85].

Additionally, the β-aryl ether bonds in lignin get cleaved when the hydroxyl group is present at α-carbon [86]. The condensation reactions in kraft-pulping account for high molecular weight fragments of lignin obtained as the product. The delignification and condensation reaction interestingly proceed through common intermediate moieties [84].

The black liquor generated from the kraft process contains condensed lignin and lignin fragment, sugars, and inorganics. The composition is directly dependent on feedstock characteristics like wood (genus and/or species) and a composition of white liquor [85]. A number of processes have been used to recover lignin from black liquor. The type of recovering process usually decides the name of recovered lignin (Table 2). The H-factor is a kinetic model based quantifier for the rate of delignification for kraft pulping (Equation (1)) [87]. It combines temperature (T) and time (t) of treatment into one quantifier.

(1) $$H=\int_{0}^{t}exp\left(43.2-\frac{16115}{T}\right)dt$$

**Figure 6:**
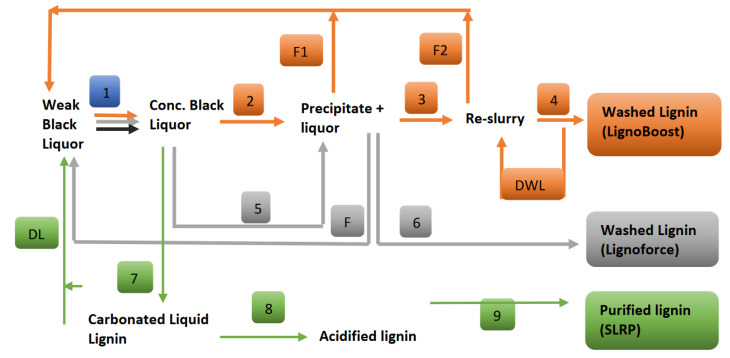
Representative diagram of lignin recovery by different processes. 1-evaporation and concentration: F, F1 and F2-filtrates; 2-pH adjustment to 9.5 using CO_2_ and aging to flocculate followed by filtration; 3-Filtrate re-slurried to pH 2 using H_2_SO_4_; 4-Filtration and washing using wash liquid (W.L.); DWL- Displacement wash liquor; 5-Oxidation using O_2_ followed by acidification using CO_2_ (pH10), coagulation and filtration; 6-Precipitate/lignin cake washing with dilute H_2_SO_4_; 7-Carbonation using CO_2_ and settling (pressurized); 8-Acidification using H_2_SO_4_ and brine (pressurized); 9-Washing for ash removal (water); DL-depleted liquor.Arrows represent reaction steps (Orange-Lignoboost, Grey-Lignoforce, Green-SLRP).

Acid precipitation is a commonly used method for lignin recovery. It involves protonation of phenols in lignin by reducing the pH in the range of their pK_a_ values (affected by temperature, ionic strength of the solution, and molecular structure of lignin), leading to the conformational changes in lignin molecules (reduction of electrostatic repulsion between lignin molecules and finally reducing their hydrophilicity) and finally flocculation [88,89]. As the molecular weight and the ratio of phenol to aromatic unit play a role in lignin solubility, the molecular weight distribution in precipitated lignin contributes to the yield [90,91,92]. Based on acid precipitation, LignoBoost technology was developed in 2002 to maximize lignin isolation and optimize salt-removal [36]. It started by using CO_2_ to adjust the pH (around 9.5), followed by aging to flocculate and first-filtration. In the next step, recovered lignin (from the first step) was re-slurried, and pH was adjusted to 2 using H_2_SO_4_. In the final step, lignin was isolated by filtration, and filtrate liquid was displaced by dilute H_2_SO_4_ (Figure 6). In further improvement to the technology, the lignin germ particles were added to improve flocculation [93]. Based on LignoBoost technology, another product by Domtar Plymouth Mill (North Carolina, USA) was marketed as Bio-Choice lignin.

In Kraft black liquor, poorly water-soluble and odor-causing totally reduced sulfur (TRS) compounds like hydrogen sulfide, methyl mercaptan, dimethyl sulfide, and dimethyl disulfide can be found. Most of these compounds can be removed by washing. However, oxidizing them can also be an alternate solution. Based on this approach, another method (LignoForce process) was developed [94]. It started with oxidation with O_2_ at 75 °C to reduce sulfide concentration (TRS compounds converted into sulfate, sulfonate, and sulfone [95]), followed by pH acidification using CO_2_ (pH 10). The slurry obtained in the previous step was coagulated (at 60–70 °C) to increase the particle size and finally filtered (Figure 6). In another patent, an apparatus integrating pressurized CO_2_ acidification with turbulent mixing in one tubular reactor with a rapid release at the exit was reported [37]. It leads to the reduction of dwell time of lignin (less than 300 s).

In another approach, a sequential liquid-lignin recovery and purification (SLRP^TM^) technology was suggested. This technology is based on the carbonation-driven formation of the lignin-rich liquid phase from black liquor [96]. Following phase separation, lignin is precipitated from the lignin-rich liquid phase using acidification by H_2_SO_4_ (sulphuric acid). The process reported recovery of H_2_S in white liquor and the reduction of CO_2_ consumption by 30% [39,97].

## 3. Organosolv Lignin

**Figure 7:**
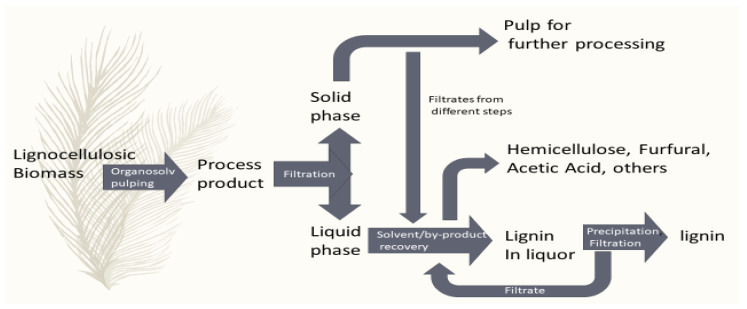
Representative flow diagram of general steps of Organosolv lignin production.

As evident from the name, the Organosolv pulping (Figure 7) involves a mixture of solvent/solvents with/without catalyst and water as a processing medium. The primary process involves delignification by lignin solubilization [34]. The suggested classification of Organosolv pulping solvent includes low-boiling-point solvents (mainly alcohols), high-boiling-point solvents (glycols and glycerol), organic acids, ketones, and others [98].

The ethanol-based process called alcohol pulping and recovery (APR) process utilizes an ethanol/water mixture (40–60% *v*/*v*) at 180–210 °C and 2–3.5 MPa [43]. The solvents are recovered using flash distillation, vapor condensation, and vacuum stripping [99]. This process was later named as the Alcell process in 1987. The underlying mechanism mainly involves the cleavage of α-O-4 ether linkage; however, other reactions (for example, β-O-4 cleavage) come into play with the addition of catalysts, other chemicals, and severity [100]. The condensation reaction can occur between the α-position of the side and 6-position of another aromatic ring via alkoxylation [101,102].

As compared to the kraft process, organosolv pretreatment involves pH (catalyst) and additional elements in addition to the time (t): reference temperature (Tr, 100 °C) and temperature (T). Therefore, to address these three factors’ contributions, a combined severity factor (CSF) was introduced (Equation (2)) [103].

(2) $$CSF=\log\left[t.\exp\left(\frac{T-Tr}{14.75}\right)\right]-pH$$

It has found applications mainly in process optimization. Traditionally applied in hot water pretreatment and steam explosion, it has applications in optimizing acid-catalyzed ethanol-based organosolv pretreatment. Although its values depend on the nature of the lignocellulosic feed, it is typically selected in the range of 1.0 to 2.5 to get good cellulose recovery and improved enzymatic digestibility. The alkaline sulfite anthraquinone with methanol (ASAM) process involves alkaline sulfite and the anthraquinone and methanol, which act as catalysts to enhance the chemical penetration and improved delignification of the biomass [104]. The anthraquinone accelerates the delignification rate and acts as a polysaccharide stabilizer. The methanol enhances chemical penetration and improves the solubility of anthraquinone [105,106]. The Formico process by the Chempolis company involves formic acid and/or acetic acid based solvents. The process starts with formicodeli process to fractionate cellulose, hemicellulose, and lignin, and is followed by fractionation; cellulose is used for pulp production, and lignin is separated in the next step. The residual hemicellulosic content is used for acetic acid and furfural production using the formicopure process [107]. The Organocell process (single-stage process) uses 25–30% methanol in cooking liquor. The functions of methanol (penetration improvement) and anthraquinone (stabilization of polysaccharides and lignin dissolution) remain the same as in the ASAM process [108,109]. Methanol is recovered by evaporation, and lignin is recovered by lowering the pH. The organic acid-based MILOX method makes use of peroxyformic acid or peroxyacetic acid as the cooking medium. They are prepared by an equilibrium reaction between hydrogen peroxide and formic acid or acetic acid. These two chemicals are selective for delignification as they do not react with cellulose and other polysaccharides. A three-stage cooking process is used to minimize peroxide consumption. First, acid cooking in the presence of small amounts of peroxide (80 °C); second, refluxing in formic acid without peroxide (100 °C), and finally oxidative peroxyformic acid cooking (80 °C) [110,111]. Acetic acid is favored as a delignification/cooking medium due to its lower molecular weight and high reactivity [111]. The Acetosolv process (the catalyst-free process is called Acetocell) utilizes 0.1–0.2% hydrochloric acid-catalyzed acetic acid for delignification at 110 °C and atmospheric pressure or higher [44]. The process developed by Compagnie Industrielle de la Materière Végétale (CIMV) utilizes a mixture of acetic acid, formic acid, and water (30:55:15, *v*/*v*/*v*) at 95–110 °C for 3.5 h, avoiding the degradation of hemicellulose (xylose) [48,49,112,113]. The lignofibre (LGF) process employs organic solvent (ethanol or acetic acid) and phosphinic acid (a strong reducing agent). The acetic acid LGF pulping showed improved delignification as compared to the mineral acid-catalyzed acetic acid process [114]. The phosphinic acid in the acetic acid LGF process supports acidolysis via phosphinic acid esterification. Furthermore, it can also protect lignin against condensation reactions taking place in an acidic medium [115]. Another ethanol-based technology (based on the Alcell process), called Lignol biorefinery technology, employs ethanol (35–70% (*w*/*w*)) with liquor to solids ratio of 4:1 to 10:1 (*w*/*w*) at the pH (3.8–2.0), with temperature (180–195 °C) and cooking time of 30–90 min [116,117]. These conditions result in lignin hydrolysis with lower-molecular-weight fragments dissolved into aqueous ethanol-based liquor. The lignin is further recovered by a pH change induced precipitation followed by filtration, washing and drying [118].

The Bloom process is based upon protection chemistry. The formaldehyde is used as a biomass protective reagent (prevents the formation of 1,3-dioxane structures with lignin side-chain hydroxyl groups) to limit condensation reactions between lignin and sugars [119]. As a result, near theoretical yields of guaiacyl and syringyl monomers, could be obtained by the hydrogenolysis of a lignin fraction (47 mole% of Klason lignin for beech and 78 mole% for a high-syringyl transgenic poplar). These yields were 3–7 times higher than that without formaldehyde [43,79]. American Science and Technology (AST) corporation developed a technology involving lignin solvent comprised of water, an acid (acetic acid and/or sulphuric acid), and a lignin dissolving compound (butanol/butyl ester and furan). The lignin solvent forms the circulation solvent after the addition of biomass that is later fractioned into various components [57]. The modification of the same method involved oxidant (gaseous O_2_/hydrogen peroxide) as an additional component leading to the development of an oxygen assisted Organosolv process [58]. Under these conditions, the lignin is dissolved in the organic solvent, and hemicelluloses are used to produce more organic solvent. Organic solvents are collected by separating water from the liquor, and the lignin is precipitated by a change in pH, heated, and filtered.

## 4. Lignosulfonates

**Figure 8:**
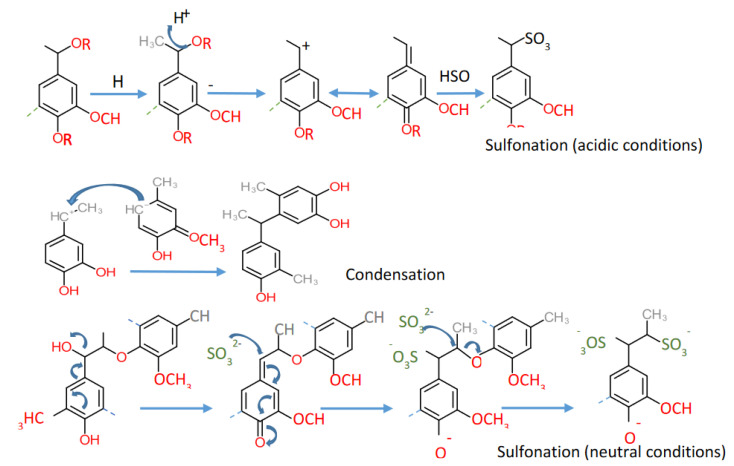
Reactions occurring during Lignosulfonate production [124].

With the annual production of lignosulfonates reported around 1.8 million tons [120], lignosulfonates contribute a significant share to total technical lignin production annually. Lignosulfonates are obtained from sulfite pulping, wherein sulfite or bisulfite is used to digest the biomass over a wide pH range. The solubility of sulfite salt is dependent on the pH of the solution; therefore, based on pH, the cation can be Ca^2+^, Mg^2+^, Na^+^, and NH_4_^+^. Calcium can be used in acidic bisulfite pulping (precipitates at pH ≥ 3), magnesium at pH ≤ 5, and at higher pH, only ammonium and sodium are plausible as cations [121,122]. The choice of cation also affects the properties of the final lignosulfonates. For example, sodium cations produce extended lignins (suitable for dispersant applications), while calcium cations result in compact lignin chains [123]. The sulfite process can be classified as an acid sulfite process (pH 1–2), neutral sulfite process (pH 5–7), and alkaline sulfite process (pH 9–13.5). In the acidic sulfite process, the primary reactions involve forming an α-C cation from the cleavage of α-O-4 ether linkage, followed by sulfonation and acid hydrolysis (Figure 8). In other words, the loss of the hydroxyl group or cleavage of the ether bond leads to the formation of a resonance stabilized benzylic cation. It is followed by the covalent bonding of sulfur as a sulfonate group (sulfonate groups attach to α-carbon) [123,124]. This sulfonate formation is responsible for lignin solubilization. Along with sulphonation, the condensation reaction involving the benzylic carbon of a molecule and another electron-rich carbon atom (due to the presence of benzylic cation) may also take place. An antagonistic reaction of the sulphonation occurs at the α-position. The specific characteristic of lignosulfonate is its high solubility at a wide range of pH.

The resulting products of the sulfite process have two main ionizing groups, that is, sulfonates (pKa 2) and the phenolic hydroxyl groups (pKa around 10). This low pKa value of the sulfonate group is a major contributing factor for increased solubility of lignosulfonates [125]. In the neutral sulfite process, the sulfonation reaction proceeds with phenolic type substrates (in contrary to an acidic condition wherein phenolic and non-phenolic type substrate take the benzylic cation route). The electron-withdrawing effect of an α-sulfonic group of the β-O-4 moiety favors the nucleophilic attack by sulfite on the β-carbon and therefore leading to the sulfitolytic cleavage of β-aryl ether bond (Figure 8).

The primary components of spent sulfite liquor (SSL) are lignosulfonate, acetic acid, sugars (from hemicellulose), inorganics, and derivatives of sugar dehydration. In the context of sulfite pulping, the multiproduct approach co-evolved with the concept of biorefinery. Traditionally, Howard’s process was employed to separate lignosulfonate from SSL [126]. It involved the addition of lime suspension to SSL, leading to the precipitation of Ca-salts of lignosulfonate and sulfite. The former formed a low-density colloidal phase and later settled as sediment [127]. A method to use calcium lignosulfonate to make vanillin was also patented [128]. Many developments have been made on the biorefinery concept using the sulfite process and reviewed elsewhere [129], therefore excluded in this paper.

## 5. Soda Lignin

**Figure 9:**
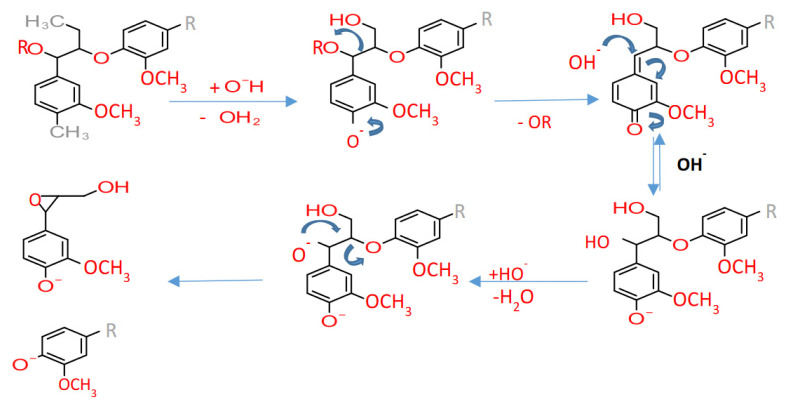
Cleavage of the α and β ether linkage during the soda/alkaline process.

The soda pulping or delignification using strong alkali (derived lignin called soda-lignin), involves heating the biomass to 140–170 °C in the presence of 13–16% alkali. However, the severe pulping conditions lead to cellulose degradation and finally, inferior quality pulp. Furthermore, to limit the cellulose degradation, anthraquinone (A.Q.) is added, and the process is called the Soda-AQ process. The mechanism of A.Q. action follows the transfer of electrons in the aldehyde groups of biomass sugars to the A.Q. molecule and the formation of carboxyl groups. This carboxyl group formation increases the stability of sugars, and ultimately the pulp yield gets improved [130]. The primary reaction in soda pulping is the cleavage of the non-phenolic β-O-4 bonds in alkaline conditions (Figure 9). The non-phenolic β-O-4 bond cleavage leads to the formation of phenolic lignin moieties and an epoxide that participates in further reactions in extreme alkaline conditions [131].

The NovaFiber process (Soda-AQ based, developed by KIRAM AB, Sweden) involves two-stage processing. First, Soda-AQ is pre-cooked to about Kappa number 60, followed by carbonate buffered oxygen assisted delignification to Kappa number 30 [63]. Another commercial producer of Soda lignin from wheat straw/annual fibers (Protobind^TM^ product family) is GreenValue SA (Switzerland) with 10,000 tons per year. The biomass is digested using aq. sodium hydroxide to produce high quality sulphur-free soda lignin [65]. Another major producer of soda lignin (Polybind^TM^ Product Family) is Northway Lignin Chemical (North America). The lignin is produced by cooking woody biomass in aq. sodium carbonate under pressure [65].

## 6. Hydrolytic Lignin

**Figure 10:**
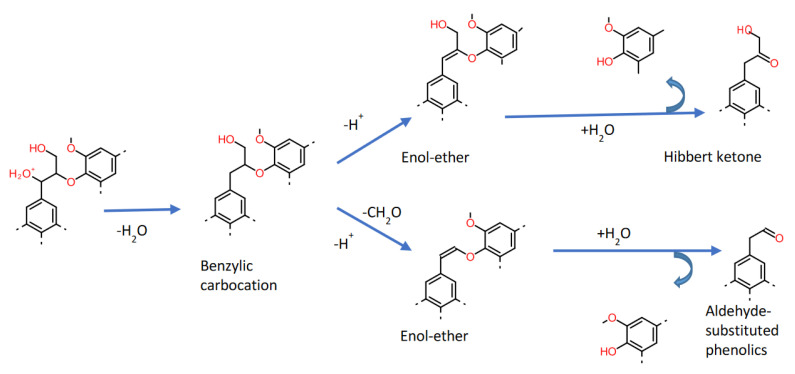
Acidic hydrolysis of lignocellulosic biomass [134].

The acid-mediated hydrolysis of lignin is among the classical approaches of biomass delignification. The acidic hydrolysis does not affect (or promote) lignin solubility, unlike alkaline treatment [132]. In acidic conditions, the reaction starts with β-O-4 bond cleavage via protonation and is followed by the dehydration of the benzylic-hydroxyl group, leading to the formation of benzylic carbocation (Figure 10). This carbocation can follow either deprotonation or Cβ–Cγ bond cleavage to form an Enol-ether. These enol ethers on subsequent hydration of the Cα=Cβ bond generate a hemiketal or hemiacetal, that leads to the carbonyl group formation on β-carbon upon β-O-4 cleavage [133]. The enol-ethers formed by deprotonation, on subsequent hydration lead to the formation of Hibbert ketones (ketone substituted phenolics with C_3_-side chain); however, enol-ethers formed by Cβ–Cγ bond cleavage upon hydration lead to the formation of aldehyde-substituted phenolics with C_2_-side chain (one carbon lost as formaldehyde). Additionally, benzylic carbocation, aldehyde-substituted phenolics, formaldehyde, and Hibbert ketones undergo repolymerization reaction leading to the condensed lignin formation [134].

Acid hydrolysis can be achieved by concentrated acid hydrolysis (e.g., 40–70 % HCl) at room temperature or dilute acid hydrolysis (<10% HCl) at high temperatures (<373 K). The Bergius–Rheinau (B.R.) process, using concentrated HCl at a low temperature, produces water-insoluble, highly condensed lignin that is difficult to valorize [68]. The B.R. process-based pilot plant has been run by HCL Cleantech (later Virdia and now Stora Enso) in Danville, USA, since April 2012. Avantium is developing a process based on the B.R. process. They have also developed a sugar/acid and lignin/acid separation technology [69].

### Technical Lignin Based Nanoparticle Synthesis: Potential and Practicability

The commercially available technical lignin (from paper mills) constitutes a regular source of lignin supply. Therefore, for the commercial success of lignin valorization, technical lignins based solutions are inherently favored [24]. Several companies across the globe are already marketing technical lignin-based commercial products. However, these products only utilize the coarse properties of the lignin for mid-to-low value applications. Lignin undergoes a significant transformation in its structure and properties during the various steps of biomass delignification [18]. As seen in Table 3, technical lignins represent a complex of protolignin derived lignin fractions, sugars, and inorganics. Due to qualitative and quantitative variation in the characteristic features of the lignin (even by the same method), the representative data in Table 3 are chosen only from one source. As seen in Table 3, the variation in carbohydrate contents, lignins (acid-soluble and insoluble), ash, hydroxyl groups number, number of average molecular weight (M_N_), weight average molecular weight (M_w_) and polydispersity Index (PDI) can be clearly seen [135]. This variation creates a significant bottleneck and must be accounted for while developing self-assembled nanostructures [136].

For nanotechnology assisted commercially-viable and high-value applications of lignin, a few things can be highlighted:

1. The nanomaterials and technologies should be developed without any significant chemical modification, as it might impinge a negative effect on cost competitiveness and scalability of the final product.

2. Technical lignin cannot be manipulated or modified at the molecular level as effectively as the other natural or synthetic polymers.

3. The developed application should be able to use hundreds of tons of technical lignin that is commercially available.

4. The property enhancement or modification achieved by change in size or morphology (in the case of a nanoparticle) should yield sufficient revenue for the acceptance of the technology as compared to micronized particles that are already commercially available.

5. Ideally, the new product/technology should be developed considering the existing production line and should require no or minimal modification.

6. The environmentally benign nature of lignin must be retained and by-product handling must be carefully thought through (ideally close-loop).

The technical lignin self-assembly should generally follow the common-trend of the molecular behavior as reported in the various studies [1,24]. The non-covalent forces like π–π stacking (due to the flat disc-shaped structure of lignin molecule), hydrogen bonding, chain entanglement, van der waals forces, and hydrophobic interactions are common forces for driving the self-assembly of lignin. The J type molecular assembly (a case of π–π stacking) was confirmed for sodium lignosulfonate [137]. However, the sulfonate group’s role cannot be ignored at the molecular level, and detailed studies in this regard are still missing. For example, lignosulfonates’ water solubility in contrast to the other technical lignin and presence of sulfonate groups are much more than the commonly used termed “water-soluble lignin”. This difference gets further enlarged during molecular-level/nano-scale modifications that are usually required in lignin-based nanotechnology.

For understanding the different scales of technical lignin self-assembly, different studies have been done. These studies start with a solution structure [138], subunit assembly [11,139], and finally, the nanostructure formation. Yang et al. (2018) studied the solution structure of Kraft lignin dissolved in E.G. and DMSO. They verified the role of hydrogen bonds for the dissolution of Kraft lignin in DMSO and E.G. It was found that hydrogen bonding was not necessary for Kraft lignin dissolution in DMSO. However, hydrogen bonding was crucial for lignin dissolution in EG [138]. These studies are vital for the development of lignin nanoparticles-based formulations for different industries (Table 4).

The lignin-based dispersants, binders, and processing-aids for agricultural applications are among the primary applications of lignosulfonates. These applications in agriculture include seed coating, granulations, wettable powder, and suspension dispersants [166,167]. A few examples are Borresperse CA/NA/3A, Norlig 11D, and Yiltrazine NA from [168] Borregard Lignotech. The other applications include soil amendments, complexing agents, granulating aids, and dispersing agents. In soil amendment applications, the lignin-based agricultural formulations work by complexing the nutrients in a readily available form to the plants [168]. For example, lignin-based BorreGRO CA from Borregard Lignotech is a Ca-rich molecule; it can be directly applied to the growth zone or via watering systems. The complexing system can also be used for foliar micronutrient delivery, mostly suitable for high-value crops like vineyards and ornamentals plants, for example, Marasperse AG powder. Other applications include fertilizer binders that provide suitable mechanical properties to fertilizers and granulated nutrient products along with dust reduction [28].

The commercial applications of technical lignin in animal feeds can be found in Aquafeed and fishery, pig feed, poultry, and ruminant feed [61,169]. A commercial product SoftAcid Aqua E is a combination of acetic acid and lignosulphonic acid. It inhibits bacterial growth and delays the degradation of fish. It claims to have reduced corrosion and safer handling as compared to the acetic acid for fishmeal preservation. The product SoftAcid is also sold for reduction for *vibrio bacteria* in shrimp farming. Another lignin-based product is PELLTECH^TM^; it shows a dual role of pellet binder and die lubricant for animal feed formulations. It has also been suggested for utilization in wood pellets [170]. In lignin-based pig feed products, the commercial products include pelleting aids, SoftAcid based pig feed bacterial growth inhibitor with reduced corrosion, SoftAcid based piglet feed to improve digestibility and antibacterial effect, and inhibitory effect on yeasts and molds (wet feed) and for drinking water (pH control with reduced corrosion). In poultry feed, along with a pelleting aid, applications include SoftAcid based antibacterial and antioxidant products (to improve protein and nitrogen uptake and thereby reduce ammonia emissions) finally for drinking water solutions similar to pig feed solutions.

In the ruminant feed category, SoftAcid based solution is suggested for controlling the unwanted increase in total mixed ration (TMR) temperature (by achieving optimal pH to balance action on molds and yeasts (optimal inhibition at pH around 3) as well as on the bacteria [170] (increased inhibition with a decrease in pH)). Lignobond DD^TM^ is a lignin-based binder for animal feed blocks to provide the right value of hardness for immediate and delayed (deterioration resistant due to inclement weather) consumption. Modified-lignin-based battery additives to extend battery life, improve cold-cranking capabilities, and increase reserve capacity have been developed [171]. A modified-lignin based product, the vanisperse^TM^ product family, acts by promoting the development of fine crystal sponge lead upon forming and later preserving this high surface structure by preventing coarsening of crystals upon cycling. Additionally, it prevents the formation of a non-conducting film of lead-sulfate. In Dyestuff industry dispersants, similar to the carbon black dispersion stabilization, the lignin-based have an advantage in dyestuff stabilization such as improved milling economy, a wide range of heat stability, controlled fiber staining, no azo reduction, decreased viscosity of final formulation, and the property of being environmentally benign [172].

In the carbon black industry, the application of lignin aq-dispersion stabilization is not new [33,173]. Lignin-based commercial suspension stabilizers for carbon black are available in the market (Vanisperse CB^TM^ and Marasperse^TM^). These products help by speeding up the milling process, increase loading, and finally stabilize the dispersion. These products adsorb onto the black and stabilize the dispersion by the steric and electrostatic mechanism. Typical applications of carbon black dispersions include conductive coating, water-based inks, and textile printing. In ceramics industry also, the applications of lignin have been widely studied [174,175]. A lignin-based commercial product, the Biokeram^TM^ family of products can be used in clay replacement, reduction of water consumption, reducing clay particle recirculation, power consumption, reduced edge erosion, chipping and cracks in pressing, reduced cracks and chipping in transportation and drying, and finally improved cracks in kiln. In building and construction related formulations, the lignin-based additives help in maintaining the rheological properties of grouts, mortar and concrete in the desirable range [176,177]. They help by improving strength, cost saving and increasing workability. The common trade names from Borredgard lignotech include—Norlig^TM^, Borresperese^TM^, and Wafaex^TM^. Additionally, BorreGYP^TM^ is another lignin-based formulation marketed as a water reducer and processing aids in gypsum board manufacturing industry as a green alternative to the synthetic-chemical based products. Another application of lignin-based products is in blending and part substitution of oil-based phenolic resins (also called resin extenders). They can work as extenders for finished resins, cutting agents after the pre-condensation step, and co-reactants during resin synthesis. Furthermore, applications of lignin road stabilization and dust control have also been reported [178]. They act by interacting with soil particles and holding them at the surface and finally reducing any dust generation. The lignin-based commercial products Dustex^TM^ and Norlig^TM^ have been used for this purpose.

Lignin based emulsion stabilizers have been marketed for application in asphalt and heavy bitumen emulsions, emulsions for use in paper sizing, wax and oil emulsions, industrial cleaners, and metalworking fluids (MArasperse ES-1^TM^, Borresperse NA ^TM^, Ufoxane 2 ^TM^, Ultrazine NA ^TM^, Wanin 734 ^TM^). In the oil and gas industry, the lignin-based products act as cement retarder additives (extend pump time and allow the cement to set at the desired location), fluid loss control additives, drilling mud thinners, and H_2_S scavengers [179,180]. A complete series of Biodrill^TM^ series for low temperature to high temperature (50–150 °C) cement retarders have been marketed by Borregard lignotech. Lignin-based fluid loss control additive agents decrease filter cake’s permeability and prevent the migration of drilling mud into a formation-producing zone. The filter cake thus formed prevents the migration of formation fluids in drilling fluids. The drilling mud thinners are used to de-flocculate bentonite clay systems, reducing shear rate viscosity and gel strength [170]. Another commercial product used as an H_2_S scavenger works by combining metal salts with lignin-based chelating agents and therefore solubilizing H_2_S scavengers for fresh and saline water drilling systems (Biodrill^TM^ Green Scav Zn/Fe). The other industrial application, where lignin can be applied, includes liquid and powder blending, liquid blending and spray drying, industrial cleaners, water repellent, and industrial binder [181].

## 7. Conclusions

The technical lignin shows a wide range of variation in its structure and characteristics originating from its source and extraction method. The behavior and properties of technical lignin are a coarse level generalization of our understanding. The molecular level approach makes it described by case specific properties rather than “lignin properties”. The gap between classical lignin microparticles and nanoparticles applications can be observed between reported lab-scale nanoparticle application and lignin-based commercial products targeted to the different industries. It can be seen that lignin-based marketed products cater to different industries as compared to the lignin-nanoparticles with some degree of overlap, and they utilize a “consumption-centric rather than unique application-centric” approach. Some applications like nanocomposites can be taken as a functional upgrade of classical materials. However, the high-value application (like biomedicine) of lignin seems far from reality for the industrially available technical lignin due to cytocompatibility and cytotoxicity reasons. It is analogous to the case of biomass-based nanocellulose that is relatively inert (except bio-cellulose of microbial origin) and its challenges are to be a regular part of commercial drug delivery formulations. Additionally, it also needs to be kept in mind that lignin itself is not inert to the biological system and has shown biological systems activity. The future of lignin for high-value applications will not offset it from existing paper mills but perhaps special-purpose biorefineries.

## Figures and Tables

**Table 1:** Lignin from different sources and different chemical bonding [18].

Bonding	Softwood	Hardwood	Grassess
β-O-4 linkage	45–60	60–62	74–84
β-5 and α-O-4 Linkage	9–12	3–11	5–11
β-β and α-O-γ Linkage	2–6	3–12	1–7
(5-5), α-O-4, and β-O-5 linkage	5–7	<1	--
β-1) and (α-O-α) Linkage	2	2	--
4-O-5 linkage (E)	1–9	1–7	--

**Table 2:** Technical lignin and their applications.

Technical Lignin	Remarks	Remarks and Applications
Kraft	Discovered by Carl F, Dahl in 1879 [26], sulphur content- 1–3%	Fertilizers and pesticides [27,28], Carbon fibers [29], Blend with thermoplastics [30], Resins [31], Ion-exchange resins [32], Activated carbons [33], Preparation of low molecular weight compounds [34]
Indulin	Based on acid precipitation. Marketed since the 1950s by Ingenivity, Virginia, USA. Classical technical Kraft-lignin in the market [35].	
Lignoboost	Developed by Inventia and Chalmers Technical University in 2002 [36], Nordic Paper Backhammer (8000 tons per annum) in 2015 [37]	
Bio-choice lignin	In 2013, Lignoboost by Domtar Plymouth Mill, North Carolina, USA, Marketed as Bio-choice lignin.	
Lignoforce	Developed by FPinnovations group and NORAM, Hindon pulp mill Alberta, Canada (30 tons per day) [38].	
SLRP	Sequential liquid-lignin recovery and purification [39].	
Organosolv	Discovered in 1968 by Kleinert [40], sulphur-free.	High number of reactive sites (supports further modification), low mol weight (not material of choice for binder and adhesives) and high purity. Additive to inks, coatings and paints [41]
Formico process	Formic acid and/or acetic acid based method [42].	
Alcell process	Alcohol based pulping and recovery (APR) process before 1987, later named as alcell process [43].	
Acetosolv and Acetocell process	Acetic acid based pulping without (Acetocell) or with catalyst (Acetosolv) process [44].	
Formacell process	Based on Acetosolv (mixture of formic and acetic acid) [45].	
Organocell process	Sodium hydroxide, methanol and catalytic amount of anthraquinone as cooking medium [46].	
ASAM process	Alkaline Sulfite Anthraquinone and Methanol based cooking medium [47].	
CIMV process	Mixture of acetic acid, formic acid and water as cooking medium [48,49], trademark-Biolignin.	
Lignofibre process	Organic solvent (Ethanol or acetic acid) with phosphinic acid [50].	
Milox Process	Peroxyformic and peroxyacetic acid based process [51,52,53].	
Lignol technology	Derived from Alcell process, Ethanol-based process [54,55].	
Bloom Process	Formaldehyde-based protection chemistry [56].	
AST process	Acid, lignin dissolving solvent, water with or without oxidant [57,58]	
Soda lignin	Sulphur-free, [59].	Reduced toxicity and increased biocompatibility Phenolic resins [60], animal feed [61], dispersants [62]
NovaFiber Process	Soda-AQ precooking followed by carbonate buffered oxygen delignification [63].	
Protobind products	Aq. NaOH based method, mainly non-wood/grass based, Tradename for the family of lignin products by Greenvalue [64].	
Northway lignin chemicals	aq. sodium carbonate treatment of woody biomass under pressure [65,66].	
Acid Hydrolytic Lignin	Developed as pretreatment method, sulphur may be present or absent.	Good sorption properties, used as sorbants [67]
Bergius-Rheinau Process	Concentrated hydrochloric acid based method, used by HCl cleantech (later Virdia Inc and now Stora Enso) [68].	
DAWN technology by Avantium	Bergius-Rheinau Process based method developed by Avantium [69].	
Lignosulfonate	Sulphite process, sulphur- 3.5–8.0%.	Unique colloidal properties due to variety (hydroxyl, carboxylic and sulphur containing) of functional groups. Binder and drilling agent [70], animal feed [71], glue and particles boards [72]
Domsjo Lignin. Now Aditya Birla group	Sodium lignosulfonate [73].	
Borregaard Lignotech	Calcium lignosulfonate [74,75,76].	
La Rochette venizel, now Saica	Ammonium lignosulfonate [77,78].	
Nippon paper chemical	San X^TM^, Vanillex^TM^, and Pearllex^TM^ (Ca, Na, Mg salts) [79].	
Cartiere Burgo	Lignin solubilized as calcium salt of sulphonic acid from Norway spruce, commercial names-Bretax and Sartex [80].	
TEMBEC, now Rayonier Advanced Materials	Ammonium and sodium lignosulfonates, commercially available as ARBO- range of products.	
Others		Green method
Ionic liquids, Molten salt hydrates,	Several patents exist [81,82], Industrially produced material absent	

**Table 3:** Variation in chemical composition and characteristics of different technical lignins [135].

Technical Lignin Type	Kraft (Indulin)	Soda (P1000)	Organosolv (Alcell)	Organosolv (Wheat Straw)	Organosolv (Poplar)	Organosolv (Spruce)
Chemical composition: weight percent per unit dry weight						
Arabinan	0.1	0.2	<0.1	0.1	<0.1	<0.1
Xylan	0.6	1.5	0.1	0.2	0.2	0.2
Galactan	0.6	0.2	<0.1	<0.1	<0.1	<0.1
Glucan	0.1	0.5	0.1	0.2	0.1	0.3
Mannan	<0.1	<0.1	<0.1	<0.1	<0.1	0.6
Sum	1.4	2.4	0.2	0.5	0.3	1.1
Ash	2.6	2.5	<0.1	<0.1	<0.1	<0.1
Sulphur	1.7	1.1	0.0	0.1	0.0	0.0
AIL	90.3	85.1	94.3	94.1	94.3	95.5
ASL	1.9	5.4	1.9	0.9	1.6	1.8
Hydroxyl group content:						
Aliphatic-OH	1.79	1.26	1.04	1.27	0.80	1.43
5-OH	1.31	1.73	1.68	1.24	1.89	1.21
G-OH	1.30	0.73	0.58	0.92	0.58	1.44
p-hp-OH	0.16	0.40	0.11	0.38	0.18	0.08
Total Ar-OH	2.77	2.86	3.30	2.54	2.59	2.73
Molecular weight:						
M_w_ (g mol^−1^)	4290	3270	2580	1960	2180	2030
M_N_ (g mol^−1^)	530	620	600	450	570	420
PD	8.1	5.2	4.3	4.4	3.8	4.9

**Table 4:** Different technical lignin-based nanoparticles and their industrial applications.

Technical Lignin	Reported by	Application
Alkali Lignin	Wang et al. 2019 [140]	Cosmetics
	Yin et al. 2018 [141]	Wastewater treatment
	Azimwand et al. 2018 [142]	Wastewater treatment
	Dai et al. 2017 [143]	Biomedicine
	Li et al. 2017 [144]	Biomedicine
	Siddiqui et al.2017 & 2020 [9,10]	Biomedicine
	Mishra and Wimmer, 2017 [136]	Coatings
Kraft Lignin	Sipponen et al. 2017 [145]	Emulsion stabilization
	Sipponen et al. 2018 [146]	Biocatalyst
	Mattinen et al. 2018 [147]	Biomedicine
	Mattinen et al. 2018 [148]	Biomedicine
	Gonzalez et al. 2017 [149]	Wastewater treatment
	Figueiredo et al. 2017a,b [150,151]	Biomedicine
	Lievonen et al. 2016 [152]	Novel Method
	Silmore et al. 2016 [153]	Dispersants
Organosolv	Liu. et al. 2019 [154]	Biorefinery
	Tian et al. 2017 [155]	Nanocomposites
	Tian et al. 2017 [156]	Nanocomposites
	Gutiérrez-Hernández et al. 2016 [157]	Cosmetics
Hydrolytic lignin	Zikeli et al. 2019 [158] (Acid)	Paint and coating
	Gong et al. 2017 [159] (Acid)	Enzyme immobilization
	Yu et al. 2018 [160] (Enzymatic)	Activated carbon
Soda lignin	Xing at al. 2019 [161]	Packaging, Agriculture
	Xiao et al. 2019 [162]	Wastewater treatment
	Chen et al. 2018	Biomedicine
	Yang et al. 2018 [163]	Biomedicine
	Yang et al. 2018 [164]	Coatings
	Juikar and Vigneshwaran, 2017 [165]	Biomedicine
	Gutiérrez-Hernández et al. 2016 [157]	Cosmetics
